# Preliminary Rashes in Measles

**Published:** 1900-09-01

**Authors:** 


### PRELIMINARY RASHES IN MEASLES.

Dr. Thursfield,[1] of the Hospital for Sick Children, draws attention to the preliminary rashes which are apt to occur during the so-called incubation stage of measles. In a small outbreak in one of the wards at the Children's Hospital, out of the seven children attacked five had the preliminary rashes, which were so obvious and striking as to appear worthy of more consideration than they have received at the hands of most writers. Dr. Thursfield says that he believes these rashes are familiar to the medical officers of the Metropolitan Asylums Board's hospitals, where they come in as cases of scarlet fever. The eruption appears to take the form of a blotchy scarletiniform, or a fine papular rash, and it occurs during the febrile period which precedes the true measles rash. In one case it is described as a brilliant scarletiniform eruption round both elbows and extending some distance up the back of the arm, while the next day the patient had a brilliant erythema which developed in patches over the face and trunk, the face being scarlet with a well marked "oval ring." Clearly from their showing themselves so soon after the commencement of the pyrexia, and from their character, it would be easy to mistake these rashes for that of scarlet fever, especially when, as often happens in private practice, one is forced to come to an immediate diagnosis.

[1] Lancet, Aug. 18.

